# Clinicians’ Perceptions of the Benefits and Challenges of Teleoncology as Experienced Through the COVID-19 Pandemic: Qualitative Study

**DOI:** 10.2196/34895

**Published:** 2022-02-24

**Authors:** Jordan M Alpert, Greenberry Taylor, Chelsea N Hampton, Samantha Paige, Merry Jennifer Markham, Carma L Bylund

**Affiliations:** 1 College of Journalism and Communications University of Florida Gainesville, FL United States; 2 Department of Communication Flagler College Saint Augustine, FL United States; 3 Division of Hematology & Oncology Department of Medicine University of Florida Gainesville, FL United States; 4 Department of Health Outcomes & Biomedical Informatics University of Florida Gainesville, FL United States

**Keywords:** teleoncology, telemedicine, qualitative, COVID-19, telehealth, cancer care, cancer, oncology, digital health, pandemic

## Abstract

**Background:**

COVID-19 thrust both patients and clinicians to use telemedicine in place of traditional in-person visits. Prepandemic, limited research had examined clinician-patient communication in telemedicine visits. The shift to telemedicine in oncology, or teleoncology, has placed attention on how the technology can be utilized to provide care for patients with cancer.

**Objective:**

Our objective was to describe oncology clinicians’ experiences with teleoncology and to uncover its benefits and challenges during the first 10 months of the COVID-19 pandemic.

**Methods:**

In-depth, semistructured qualitative interviews were conducted with oncology clinicians. Using an inductive, thematic approach, the most prevalent themes were identified.

**Results:**

In total, 21 interviews with oncology clinicians revealed the following themes: benefits of teleoncology, such as (1) reducing patients’ travel time and expenses, (2) limiting COVID-19 exposure, and (3) enabling clinicians to “see” a patients’ lifestyle and environment, and challenges, such as (1) technological connection difficulties, (2) inability to physically examine patients, and (3) patients’ frustration related to clinicians being late to teleoncology appointments.

**Conclusions:**

Teleoncology has many benefits and is well suited for specific types of appointments. Challenges could be addressed through improved communication when scheduling appointments to make patients aware about what to expect. Ensuring patients have the proper technology to participate in teleoncology and an understanding about how it functions are necessary.

## Introduction

Telemedicine, defined by the Institute of Medicine as the use of electronic information and communications technologies to provide and support health care when distance separates the participants [1], was not often utilized in cancer care prior to COVID-19 [2-4]. Although advocates of telemedicine called for improved access to the technology before COVID-19 [5-7], the pandemic forced health systems to rapidly adapt. For instance, a study evaluating claims data found that telemedicine utilization for office visits and outpatient care was 78 times higher in April 2020 than in February 2020 among various diseases, including cancer [8]. Telemedicine is enabled by over 90% of adults in the U.S. using the internet, although only 77% have broadband internet service at home [9]. The surge was a result of loosening regulations, which allowed for insurance coverage and reimbursements for telemedicine visits [10]. In 2021, the American Society of Clinical Oncology (ASCO) published standards and practice recommendations to ensure that clinicians effectively use telemedicine with their patients now and in the future [11]. However, the review summarized previous telemedicine studies and focused on situations when it was most appropriate to deliver care rather than how patients and clinicians interact with one another using the technology.

In cancer care, effective clinician-patient communication is particularly important because it impacts patients’ psychosocial outcomes and quality of life [12]. Prepandemic, limited research had examined clinician-patient communication in telemedicine visits. In a study consisting of interviews with oncology professionals (eg, physicians, physician assistants, and nurse practitioners) about using telemedicine, Heyer et al [13] discovered that clinicians were concerned about whether they could effectively build rapport and provide patients with the support necessary to nurture clinician-patient relationships that are integral to quality care. The study was conducted between October 2019 and March 2020, immediately preceding the COVID-19 pandemic [13]. Questions remain about whether these perceptions persisted during the pandemic, as telemedicine became entrenched in the health care delivery experience. A recent paper that surveyed both patients and cancer clinicians during the pandemic found that patients are more enthusiastic about using telemedicine than clinicians, with a greater number of responses stating that clinicians prefer in-person visits [14].

We use the term “teleoncology” in this study to refer to visits between cancer patients and clinicians conducted over videoconferencing applications, such as Zoom (Zoom Video Communications). The rapid shift during the first few months of the COVID-19 pandemic to teleoncology [15] has provided an unprecedented opportunity to understand oncology clinicians’ experiences with the technology. Research has assessed the patient perspective in cancer care, finding that patients experience technical difficulties [16] but are also largely satisfied with the encounter [17]. Thus, the purpose of this qualitative study was to describe oncology clinicians’ perceptions of teleoncology and to identify its benefits and challenges during the first 10 months of the COVID-19 pandemic.

## Methods

### Study Design

We conducted an in-depth qualitative study at the University of Florida Health Cancer Center (UFHCC). The UFHCC is a 192-bed hospital serving North Central Florida, specializing in 14 cancers, such as blood cancer, lymphoma, breast cancer, and head and neck cancer. The cancer center serves surrounding rural counties, which make up 20% of the patient population. The University of Florida Institutional Review Board (202000243) approved the study, and all participants consented to participate before interviews began.

### Participants and Recruitment

Inclusion criteria consisted of participants being clinicians (oncologists, nurse practitioners, or physician assistants) who provided care to individuals with a cancer diagnosis and were willing to participate in an interview. We sent an email and 1 reminder email to all medical and radiation oncology clinicians at our cancer center with a description of the study and a link to an online screening questionnaire. We diversified the clinician type of our sample by asking participants for referrals toward the end of the interview. Further, we used our professional networks to contact clinicians, and we posted recruitment messages to social media, accompanied with keywords targeted toward clinicians working in cancer. A total of 59 unique recruitment emails were sent between July and December 2020. Interviews were conducted simultaneously with recruitment, as the first interview occurred in July. During this time, the number of COVID-19 cases in the state of Florida peaked in October before plateauing in December [18]. Pharmaceutical companies were also beginning to seek approval for vaccines.

### Procedures

Potential participants filled out a short online form to indicate their interest and to schedule an interview. Prior to the interview, participants were provided with a statement of their rights. All interviews were completed by 1 of 3 authors (JA, CH, and CB) using a semistructured interview guide about 3 different communication topics in cancer care (secure messaging, teleoncology, and online information seeking). Questions about communication using teleoncology during the COVID-19 pandemic made up 1 of 3 sections of the interviews. Members of the research team collectively wrote the interview guide to align with our goals of understanding clinicians’ perceptions of teleoncology. The clinical member of the research team (author MJM) reviewed the interview guide before it was finalized. Specific questions included asking clinicians about the challenges they encountered in moving to telehealth to communicate with patients, its advantages/disadvantages, and what strategies were developed to facilitate telehealth interactions. Interviews were conducted using the videoconferencing software Zoom and were audio-recorded and professionally transcribed.

### Data Analysis

The constant comparative method [19] was utilized to analyze the interview transcripts using an inductive, thematic approach. Thematic analysis is a valuable method for examining the perspectives of different participants, highlighting similarities and differences, and generating unanticipated insights [20]. Interviews continued during data analysis until no new themes emerged and thematic saturation was achieved [21] through recurrence, repetition, and forcefulness of the data [22]. The second author (GT) uploaded all transcripts to Atlast.ti v. 22 (ATLAS.ti Scientific Software Development GmbH), a software management and analysis program. Two authors (GT and CB) conducted open coding using an adapted version of Strauss and Corbin’s guidelines, [23] assigning in vivo codes. Codes were collapsed into categories, after which thematic properties were identified using axial coding. For example, each participant’s interview was examined for information relevant to 1 of the posed inquires (ie, benefit or barrier) and Atlas.ti was used to assign a code. Codes were compared and combined to generate themes, which were then examined for text that conveyed similar messages, after which those were separated into their own group (i.e., property). Codebooks were developed for each research inquiry throughout the analytical process by the second author (GT) and were discussed with the senior author (CB) to refine themes and properties before creating finalized versions. The second author (GT) created analytical notes and memos throughout the analysis process, which increased the ability to identify poignant descriptions to illustrate themes and properties. This strategy was used to increase the trustworthiness of findings as well as promote transferability [24]. The senior author (CB) used the final codebooks to conduct closed coding of all transcripts, after which the second author (GT) validated the analysis. At this point, we shared the analysis with our clinician coauthor (MM) and study principal investigator (JA) for further validation of the results.

## Results

### Participant Characteristics

A total of 21 clinicians participated in the study (36% enrollment rate). Interviews averaged 44 minutes in length and resulted in 285 transcribed pages. Of the 21 participants, 13 (62%) were female; the average number of years postresidency, fellowship, or schooling among 18 (86%) participants was 8 years (range 1-33); and 3 (14%) participants were still in residency. One (5%) participant was a physician’s assistant, and another (5%) was an advanced practice registered nurse. Most clinicians (n=17, 81%) were affiliated with the UFHCC, while the other 4 (19%) were employed at cancer centers in the south, northeast, and western U.S. Most clinicians primarily worked in outpatient settings, and 14 (67%) were in medical oncology departments and 7 (33%) in radiation oncology. Each participant reported that they used teleoncology with patients during the pandemic.

Our qualitative analysis revealed a total of 6 themes: 3 (50%) themes related to the benefits of teleoncology and 3 (50%) themes about the challenges of teleoncology. We describe each theme next and include thematic properties, when present, to provide a richer description of the themes. Additional exemplar quotes associated with each theme and property are in Tables 1 and 2.

**Table 1:** Benefits of technology.

Properties (if applicable)		Exemplar quote
**Theme: teleoncology is convenient for patients**		
	Reduces in-person visits and travel	*A lot of our patients do travel very far to see us, and I think telemedicine can be used very effectively for visits where we don’t necessarily need to see the patient in person or it’s our first encounter and we want them to get more studies done before seeing us.*
	Reduces financial burden	*It cuts down on the financial burden for them and having to come in the office purely to have a discussion and then for us to tell them, “You need more imaging,” and then them having to come back . . . same for follow-up appointments.*
**Theme: teleoncology reduces the risk of COVID-19 exposure**		
	—^a^	*I think the biggest advantage is being able to keep people who are at higher risk for complications from COVID at home and out of the general public.*
**Theme: teleoncology helps clinicians to better “see” patients and family**		
	Makes patients and their environments visible	*I get to see inside their home. So, if I can tell there’s a dog in the room, I usually ask them to show me their pet. I had a patient this week walk me by phone outside into her garden to see an orchid that she had blooming . . . It’s a neat way to connect with them that we can’t do in the clinic.*
	Facilitates family member participation	*[Maybe] they can have family members present who may not otherwise be able to be present.*

^a^Not applicable.

**Table 2:** Challenges of technology.

Properties (if applicable)		Exemplar quote
**Theme: technical challenges affect the quality and effectiveness of teleoncology**		
	Internet connectivity issues	*A lot of my patients, because they're rural, don't have Wi-Fi strong enough for me to do an actual Zoom visit. It's very frustrating, because it drops so much, and it freezes. So, they've just given up, and they just come to clinic.*
	Patients’ unfamiliarity with telehealth technology	*The biggest challenge was literacy about technology. Most of our patients, sometimes you will get into the Zoom, they are not there, and they are waiting on you, [and] then they will call the clinic. I’ve been waiting on my doctor because they don’t know how to navigate it.*
**Theme: inability to conduct a physical exam**		
	—^a^	*The challenges are definitely not being able to do a physical exam because the patient is not there with you in person. You’re seeing them in their environment, sitting in a chair, but you’re not seeing them walk into the office. You can gather a lot by watching someone walk in and if they’re struggling to walk in, those types of things.*
**Theme: challenge to meet expectations about appointment times**		
	—	*I've noticed with Zoom, there's this expectation that I be exactly on time. And our clinic schedules face-to-face and Zoom all mixed in. So, by definition, I never see a clinic patient at the time of their appointment, because they're getting vitals. So now my 9:00 AM, I don't see till 9:20, but my 9:30 expects me to be on Zoom right at 9:30, and I just can't actually do it.*

^a^Not applicable.

### Benefits of Technology

#### Theme 1: Teleoncology is Convenient for Patients

Teleoncology was described by clinicians to be better for the patient than in person-visits in many ways as it removed traditional demands (eg, driving to an appointment, planning) and requirements (eg, sitting in the waiting room, around others).

##### Reduces In-person Visits and Travel

Clinicians shared that teleoncology provided patients with an opportunity to avoid physically coming to the office/clinic to meet with providers when it was not necessary. A radiation oncologist noted that telemedicine could be an effective substitute for situations such as a first visit when more tests are warranted, general consultations, or follow-up appointments. One clinician said:

[Patients] who are on routine follow-ups . . . and some that are in remission who just come in every 3 or 6 months or even annually for lab work who don't have any new physical issues, physical symptoms, any new concerns, who are doing great, they really can just go get lab work done outside . . . They don't have to come into clinic.

Participant 28

One benefit of reducing in-person visits is not needing to travel, especially for those who live far away from the hospital, such as those who live in other parts of the state or other countries. One clinician recalled an experience where they were able to consult with a patient living in another country using teleoncology:

One of the patients I saw . . . was from the U.K., and that's [teleoncology] was the only way we were going to be able to see him at that time.

Participant 59

##### Reduces Financial Burden

Clinicians also described how teleoncology reduced the financial burden of coming to appointments in person. Clinicians cited travel expenses for individuals, especially those with a limited financial budget. One clinician spoke specifically about the advantage teleoncology afforded patients with fiscal issues:

We see those low socioeconomic groups so common and people who don't have gas money. I mean, that's huge, and obviously people that travel several hours.

Participant 8

Another clinician echoed this by addressing the distance some patients are required to drive to a clinic for a short appointment, saying:

For patients who don't have a lot of money or have transportation issues, it really saves them a visit. So, if there are things that it's just a conversation, and it really doesn't require them to drive 150 miles to have a 20-minute conversation with me, I think that's a beautiful use of telemedicine.

Participant 2

#### Theme 2: Teleoncology Reduces the Risk of COVID-19 Exposure

Teleoncology made it convenient for immunocompromised patients to avoid exposure to high-risk health care areas where COVID-19 might be present and being around the public when traveling to and from appointments. One clinician described how this form of communication enabled at-risk patients (both with cancer and in remission) to stay at home, while also highlighting that they were, and most likely would be, immunocompromised to some degree.

They don't have to put themselves at risk by coming into clinic, because some of these patients, they're cancer survivors or they're cancer patients in remission and they're still at risk in terms of their immune system. To some level, they're always immunocompromised because of their treatment, so there's no reason to bring them into [the] clinic . . . So they can stay in the safety of their own home and do a quick telemedicine visit, and it's simple and they prefer that. They don't have to leave their house.

Participant 28

The level of concern patients had regarding exposure to external environments during the pandemic was also cited by participants. One clinician described patients’ concerns and mentioned the safety this form of communication afforded immunocompromised individuals:

It allowed the opportunity for patients to stay home, be safe. A lot of these patients obviously are immunocompromised, and if it’s just like a lab check, we can do that over Zoom. We don’t need to do, like, a physical exam at that point. I think it just gives the patients peace of mind. I mean, a lot of them were very nervous, understandably, to come in. So, we’re able to provide that service.

Participant 44

#### Theme 3: Teleoncology Helps Clinicians to Better “See” Patients and Family

Clinicians reported the benefit how interacting with a patient via videoconference provided them a unique opportunity to see the patient’s environment and speak with caregivers or family members who could attend the online appointment.

##### Makes Patients and Their Environments Visible

Clinicians described the importance of “seeing” patients, as opposed to only talking to them over the phone. One participant compared it to doing a home visit in that it allowed them to assess whether the patient was physically well. Another oncologist recalled how a virtual visit with a patient helped their decision making:

I had a patient who did a telehealth visit with me from her bed, because she couldn’t get out of bed. And she wouldn’t tell me that. But the fact that she did this visit with me, laid up in bed, and hadn’t gotten ready, it told me so much about what was going on with her healthwise that it was sort of invaluable information for me to make decisions. And then just seeing where they live and what their living situation is like, and you can just get so much information from a telehealth visit that you’ll never get from an in-person visit in the clinic.

Participant 2

Clinicians also said that viewing patients’ living conditions provided an opportunity to make connections and form rapport that they might not have been able to do in a traditional setting. One clinician spoke about seeing pictures and other items inside of a house and striking up conversations with the patient. They said:

It was nice to have conversations about pictures that they had in their house, or items that they had in their house that I found interesting, and it was always a nice way to get to know people on a personal level, and kind of develop a rapport with them.

Participant 13

Another recalled having a virtual visit with a patient who was outside, and noticed animals in the background, allowing them to form a connection with the patient. They said:

One of my patients did it from outside, because that was the only place he had a cell signal, and so you could see all his chickens and his pig in the background. And I have chickens, too. So, I did talk about the chickens, and I got to meet his pig, and that was just a really lovely connection that I wouldn’t have really had with the patient.

Participant 6

##### Facilitates Family Member Participation

Clinicians noted that teleoncology provided caregivers and family members who might not be able to attend in-person visits an opportunity to engage in discussions. One clinician illustrated this by saying:

It gave us a chance to get a sneak peek into a patient’s home, which we never necessarily saw before, so patients who didn’t have caregivers ever accompany them sometimes they were in the chair next to them at the table.

Participant 7

### Challenges of Technology

#### Theme 1: Technical Challenges Affect the Quality and Effectiveness of Teleoncology

Clinicians described how a variety of technical challenges hindered the ability to conduct a clinical appointment over Zoom. This included internet issues and low confidence using computer applications.

##### Internet Connectivity Issues

Clinicians described instances when a virtual visit would be interrupted due to low bandwidth or an unstable connection. Clinicians frequently expressed how low bandwidth contributed to unstable connections for patients who lived in rural areas, which resulted in dropped calls, freezing screens, and delays.

The patients that I was doing telemedicine with live in kind of rural, outlying areas, and so I found that we could get connected . . . it took a little bit of time, and then there were lots of delays. And in a couple of situations, people got cut off, and we had to log back in.

Participant 2

##### Patients’ Unfamiliarity With Telehealth Technology

Clinicians described how patients’ lack of familiarity using technology (eg, Zoom, installing applications) negatively impacted communication. As 1 clinician described:

Everybody wasn't able to use Zoom as effectively initially, and so you'd have situations where people couldn't log in, they couldn't be heard or seen because the program wasn't working correctly, and so it was just kind of frustrating some people, so they may not necessarily show up for an appointment because they don't want to have to deal with Zoom.

Participant 58

Clinicians cited patients’ age as a contributing factor to the lack of familiarity with technology. They noted that elderly patients were not always “technologically savvy” with telemedicine services, such as Zoom or online portals, as illustrated by the following recollections:

The biggest challenge is that we have, generally speaking, an elderly population of patients, some of [whom] are very tech savvy and can Facetime or Zoom or use email. But there was some disparity that was created because some patients were not used to using technology in that way.

Participant 59

I see a particular group of patients [who] are typically elderly, and might not be technologically savvy, in order to know how to access the telehealth portal. And that became a little bit challenging, and it would have to require the help of either me or my staff to get them connected.

Participant 13

#### Theme 2: Inability to Conduct a Physical Exam

Not physically being together meant that physical exams were unable to be performed. One clinician explained how not being able to conduct a physical exam was a particular challenge with cancer patients:

You have to see these patients and be able to assess their fitness for chemotherapy, and that takes the ability to actually lay eyes on them and examine them and really teach them. A lot of the things we ask of our patients are not easy requests, and it's also part of the care is also emotional support. And sometimes, that doesn't translate as well online. So in order to give the comprehensive care that they need, then visits are important.

Participant 9

Another clinician remarked about the significance of being with a patient face-to-face. Using teleoncology, the clinician acknowledged that they were unable to see the patient walk into the office. Information gathering can occur by observing if a patient is struggling to walk or by the way they position themselves on the examination table.

#### Theme 3: Challenge to Meet Expectations About Appointment Times

Without in-person visits, clinicians also described that patients’ expectations and behaviors had changed since using teleoncology services. For example, 1 oncologist said that patients expected them to be exactly on time:

Our clinic schedules [include] face-to-face and Zoom all mixed in. So, by definition, I never see a clinic patient at the time of their appointment, because they're getting vitals. So now my 9:00 A.M., I don't see until 9:20, but my 9:30 expects me to be on Zoom right at 9:30, and I just can't actually do it.

Participant 6

Other oncologists noted that when they were not on time, some patients left the videoconference. One oncologist shared how their expectations of patients waiting on Zoom were much different from the reality, saying:

I thought when we started using it like, “This'll be great when people have to wait. If I'm running late, wouldn't you rather wait in your own home, and you'll just Zoom on?” And not so much. There's not a great way to let people know how long they're going to be waiting. That system is not really well worked out, and so I think that's kind of annoying for patients. Well, and for me too. They'll Zoom on. If you're late, they're gonna Zoom off. You got to get them back. That's kind of cumbersome.

Participant 8

## Discussion

### Principal Findings

The COVID-19 pandemic led to a major shift in the way cancer care was provided to patients for a sustained period. As there have been calls for teleoncology to be more present in cancer care [5,6], it is important to understand this almost universal experience of teleoncology from the perspective of clinicians delivering care. After conducting 21 interviews with oncology clinicians about their experiences pivoting to teleoncology during COVID-19, we found that utilization of the technology has many benefits but also has several challenges to be overcome if it is to continue as a viable option for appointments and consultations. Clinicians believed that teleoncology has nonmedical benefits for patients, such as reducing travel time and expenses related to the consultation, as well as medical benefits, such as limiting COVID-19 exposure and allowing clinicians to get a better sense of the patients’ lifestyle, environment, and incorporating family members. Challenges also comprised nonmedical and medical issues. Nonmedical factors were technology related, such as problems with internet connectivity and lack of familiarity with videoconferencing technology. However, clinicians perceived shortcomings in teleoncology because they could not have physical contact with patients, which inhibited their ability to conduct a physical exam. Further, instances occurred in which patients were disappointed and frustrated that clinicians were late to the Zoom appointment.

This study adds to the growing literature on teleoncology by highlighting the perspective of oncology clinicians. The previous literature about teleoncology has focused on the experience of using the technology as a tool to reach patients in rural settings and developing countries [25-28]. Our findings align with the literature emphasizing the benefits of teleoncology to reduce travel time and costs, but in the case of COVID-19, teleoncology was mandated as the primary method of care for patients with cancer. Adoption of new technology can be slow, especially in health organizations because organizational (eg cost, complexity, impact) and individual factors (eg age, attitude) determine when and if innovations are accepted [29]. Due to the pandemic, health systems decided to universally adopt teleoncology, even though there was uncertainty among end users (ie, clinicians), otherwise known as forced adoption [30]. As a result, clinicians in our study dealt with the benefits and challenges of teleoncology concurrently, without the ability to address and fix challenges.

However, being compelled to use teleoncology pointed out a benefit that seems to be missing in the previous literature, that of the ability to “see” the patient and their family. In this case, “seeing” could mean several things: (1) viewing the patient’s health and symptoms (as opposed to telephone only); (2) observing the patient in their home environment, which further allowed for better rapport building and connection; and (3) witnessing the patient within the context of their family situation, as family members who could not normally attend were able to. Interestingly, 1 clinician discussed how teleoncology was akin to a home visit because it was an opportunity to observe the patient in their own environment. Knowledge of a patient’s physical living space could benefit clinicians in providing care [31]. In all cases, the ability to “see” had the potential to improve care for the patient by better understanding their situation.

Challenges of teleoncology were noted as including technical difficulties, which are well established in the literature. A recent study among clinicians found that poor internet connectivity is the biggest barrier to telemedicine [32]. Lack of access to technology, which enables the use of teleoncology, is also a significant issue that has implications for health equity in cancer care delivery. Compared with younger patients, older patients with cancer are less likely to have an email address or own a smartphone and are less likely to use a patient portal to communicate with their oncology care team [33]. In addition, patients faced similar hurdles as clinicians to forced adoption of teleoncology. Digital literacy—the awareness, attitude, and ability to appropriately use digital tools and facilities to identify, access, manage, and construct new knowledge and communicate with others [34]—is a major factor that has widened the digital divide. Older adults (65+ years old) have the lowest adoption rates for using new technologies [35]. However, internet adoption among older adults has risen steadily over the past decade and a half [36]. An intervention that trained older adults to use technological devices found improvement in technology confidence and a significant increase in technology use [37]. Other than an email with instructions, patients received little guidance about shifting to teleoncology.

Another challenge faced by clinicians was the inability to conduct physical examinations. Although tools such as a weight scale, blood pressure cuff, pulse oximeter, and thermometer can be administered by patients while using telemedicine, such tools are sometimes not covered by insurance and may be prone to errors due to lack of calibration and patients’ inexperience [38]. It is important for clinicians to physically examine patients, but it is not necessary for certain types of appointments. As clinicians in our study acknowledged, teleoncology is beneficial for follow-ups and instances when patients are not experiencing any discomfort.

Interestingly, 1 of the themes that emerged was a different expectation from patients about how appointment start times should be managed. Clinicians observed that patients assumed the clinician would be present at the start of the Zoom appointment, even though it is commonplace for patients to wait for the clinician during in-person appointments. Patient satisfaction is negatively impacted by longer wait times and affects perceptions of information, instructions, and the overall treatment provided by clinicians [39]. Among patients with cancer, over 80% in an outpatient oncology clinic felt that waiting for their appointment had an emotional cost [40]. Further, over one-quarter of patients suffered a major emotional impact by seeing other sick people in the waiting room [40]. Although better coordination and communication is necessary when scheduling teleoncology appointments, patients do have the benefit of waiting in their home rather than in the clinic. If patients were made aware of possible delays or received periodic updates about the status of their appointment, perhaps fewer patients would abandon the Zoom appointment. There is the potential to damage the clinician-patient relationship when clinicians are delayed. Uncertainty and lack of communication between the patient and the health care team can have negative implications, but keeping patients informed and expressing empathy are ways of improving the interaction [41,42].

### Implications of the Study

There are several practical implications from this study for those working in clinical settings as either clinicians or administrators. First, clinicians should receive training about communicating effectively with patients using teleoncology. Our study identified challenges to using teleoncology that could be remedied with slight modifications to clinicians’ behavior. For instance, patients satisfied with encounters using telemedicine appreciated relational experiences with clinicians and when an effort was made toward building a patient-centered relationship [43]. Clinicians should also look at the camera to ensure good eye contact and foster rapport and trust [44]. Training can also include how to involve family members present on-screen and methods to managing appointment times. Second, the health care team can inform patients when scheduling about what to expect before the appointment begins. Notifying patients of potential delays and having clinicians update patients during appointments while they are waiting can reduce uncertainty. While patients are waiting, health care teams can use the opportunity to emphasize the importance of health promotion through COVID-19 risk reduction by playing videos and other educational content. Lastly, it is important to ensure that patients are prepared for the appointment by testing out the technology in advance and having flexibility about what type of technology they can use. Since the Health Insurance Portability and Accountability Act (HIPAA) relaxed its guidelines for COVID-19, tools such as Apple FaceTime, Facebook Messenger video chat, Google Hangouts video, and Skype can be utilized [44]. Patients should be offered the choice of technology to use for teleoncology in order to avoid downloading and learning new applications. For teleoncology to be successful and a valid method of care delivery, ultimately, the responsibility falls on the health care system to better accommodate the technology than placing the burden on clinicians. However, the rapid increase in teleoncology visits during the pandemic has revealed that it should have a larger role postpandemic. In 2021, at least 30 states considered legislation to revise telehealth coverage standards [45]. In addition to ensuring that all patients can access teleoncology services, including telehealth as part of routine follow-up care has been recommended because it allows for efficient discussions of laboratory and imaging results, as well as side effect management [16].

### Limitations

Although we attempted to diversify our sample by recruiting clinicians from different health systems, the majority of participants were from 1 health system. Therefore, our results may not extend beyond the health system and be generalized in other contexts. There may also be the possibility of selection bias, as participants in our study volunteered. Most participants were oncologists, but understanding the experiences of other types of oncology clinicians is critical. Interviews took place toward the end of 2020 after teleoncology use spiked in the previous months. At the time of the interviews, teleoncology was relied upon less frequently. Developments related to COVID-19 have caused frequent shifts in health care protocols, which highlights the need for further research to examine the long-term implications of teleoncology.

### Conclusion

We interviewed 21 cancer clinicians during the COVID-19 pandemic to understand the benefits and challenges of using teleoncology to replace in-person appointments. The rapid adoption of teleoncology resulted in several obstacles, such as issues around internet connectivity and miscommunication about appointment times. Benefits included reduced travel time for patients and limiting their exposure to COVID-19. Clinicians appreciated the ability to learn more about patients by observing their living conditions, which provided insights into the patient’s lifestyle. Future work is warranted to explore the attitudes and perceptions of patients, along with clinicians, in various types of cancers to understand how the technology is adapted to different types of diseases. Future research should also include family members and caregivers to understand their role in the facilitation of teleoncology and how their involvement can alter depending on the type of visit.

## Abbreviations

UFHCC: University of Florida Health Cancer Center
